# 自然杀伤细胞与癌症——杀伤细胞免疫球蛋白样受体（KIR）的调控

**DOI:** 10.3779/j.issn.1009-3419.2010.07.14

**Published:** 2010-07-20

**Authors:** Amanda K. PURDY, Kerry S. CAMPBELL, 伟强 王, 书军 李

**Affiliations:** 1 Fox Chase Cancer Center; Institute for Cancer Research; Philadelphia, PA USA; 2 天津医科大学总医院，天津市肺癌研究所，天津市肺癌转移与肿瘤微环境重点实验室

**Keywords:** 自然杀伤（NK）细胞, 杀伤细胞免疫球蛋白样受体（KIR）, 先天性免疫, 癌症细胞毒性, MHC Ⅰ类分子, 造血干细胞移植

## Abstract

自然杀伤（natural killer, NK）细胞是先天性免疫效应细胞，约占人外周血淋巴细胞总数的10%-15%，主要参与免疫监视，以消除转化细胞和病毒感染细胞。NK细胞最初被界定是由于它们具有自发消除少数主要组织相容性复合物Ⅰ类（major histocompatibility class Ⅰ, MHC-Ⅰ）自身分子表达缺乏细胞的能力，即常说的“丢失自我”识别能力。NK细胞表面表达的MHC-Ⅰ特异性抑制性受体，可使NK细胞对表达MHC-Ⅰ的正常细胞耐受，此为丢失自我识别能力的分子基础。由于缺乏抑制性受体的配体，表面MHC-Ⅰ表达下调的肿瘤细胞和病毒感染细胞易受NK细胞攻击。杀伤细胞免疫球蛋白样受体（KIR; CD158）组成MHC-Ⅰ结合受体家族，对调节人NK细胞和部分T细胞的活化阈值起重要作用。KIR多样性使NK细胞具有多种功能，在此我们将综述多个水平上的KIR多样性，并诠释KIR多样性是如何影响各种疾病（包括癌症）的易感性的。我们将进一步阐述通过针对KIR进行癌症治疗的策略：利用KIR/MHC-Ⅰ配体的错配以强化造血干细胞移植的效果，以及通过阻滞KIR以增强对肿瘤细胞的杀伤力。

## 引言

自然杀伤（natureal killer, NK）细胞是先天免疫系统的淋巴细胞，在预防病毒感染和抑制癌症发展中起重要作用^[1, 2]^。NK细胞占人类外周血淋巴细胞总数的10%-15%，由于胞内表达致密的溶细胞颗粒，故称为大颗粒淋巴细胞^[3, 4]^。当遇到某些异常的肿瘤细胞或病毒感染细胞时，NK细胞就会自发激活，并释放颗粒内容物——穿孔素和颗粒酶至靶细胞^[3, 5]^。穿孔素和颗粒酶可引起靶细胞凋亡^[6, 7]^。NK细胞需一对一地识别并粘附于异常细胞以发挥细胞毒性作用^[8, 9]^。因此，人们认为NK细胞对消除单细胞肿瘤尤为重要，尤其是白血病、淋巴瘤以及正在发生转移的肿瘤细胞^[10]^。尽管NK细胞最初由其具有产生细胞毒性作用的能力而命名，但NK细胞还能产生许多细胞因子和趋化因子，尤其像γ干扰素（interferon, IFN）、肿瘤坏死因子（tumor necrosis factor, TNF）α和GM-CSF^[11]^。除了可以直接作用于肿瘤细胞和病毒感染细胞外，这些细胞因子还可以促进其它免疫细胞的分化、活化和/或募集^[12-14]^。

NK细胞对肿瘤细胞的识别基于其特有的能力：辨识并攻击细胞表面主要组织相容性Ⅰ类（major histocompatibility class Ⅰ, MHC-Ⅰ）分子表达水平低的细胞^[15, 16]^。MHC-Ⅰ分子通常表达于体内几乎所有的细胞，许多异常细胞如肿瘤细胞或病毒感染细胞MHC-1下调，这使得它们可以逃避溶细胞性T细胞的监视^[17, 18]^。但是，MHC-1的下调（自身分子的丢失）使异常细胞对NK细胞的细胞毒性作用敏感，这一过程被称为“丢失自我”识别能力。“丢失自我”识别能力的概念于20世纪80年代晚期首次被提出^[19]^，当时很难解释淋巴细胞是如何识别细胞表面标志物丢失的细胞，因为众所周知T细胞和B细胞是通过接受体内的外源性分子被激活的^[20, 21]^。自此，人们逐渐意识到，NK细胞的应答受细胞表面激活性受体和抑制性受体间的信号平衡调控，而且通过检测这些自身分子发现，可与MHC-1结合的抑制性受体是NK细胞对正常细胞耐受的关键所在^[22, 23]^。

## NK细胞应答的调控

NK细胞激活性受体主要包括自然细胞毒性受体（NCR：NKp30，NKp44和NKp46）、Fc受体CD16、NKG2D，以及激活性杀伤细胞Ig样受体（killer cell Iglike receptors, KIR）^[24, 25]^。NCR的配体最近刚被鉴定出来^[26-28]^，NKG2D可识别非经典的MHC-Ⅰ分子──MICA/MICB和ULBPs^[29, 30]^，激活性KIR似乎可识别经典的MHC-Ⅰ分子^[31]^。另一方面，CD16与IgG抗体的Fc段结合可启动抗体依赖性细胞介导的细胞毒作用（antibody-dependent cellular cytotoxicity, ADCC）^[32]^，而且CD16可使NK细胞具有识别并杀伤抗体包被的靶细胞的能力。已有研究显示，CD1有助于rituximab（利妥西单抗）和herceptin（赫赛汀）抗体在分别治疗B细胞淋巴瘤和乳腺癌中发挥抗肿瘤作用^[33, 34]^。各种协同受体（co-receptor）（如2B4、CD2、LFA-1和DNAM-1）的共同参与使激活效应进一步被放大^[4]^。通过激活细胞内酪氨酸蛋白激酶级联反应，上述所有激活性受体可促进细胞毒性作用和细胞因子的产生（图 1）^[23]^。

**1:**
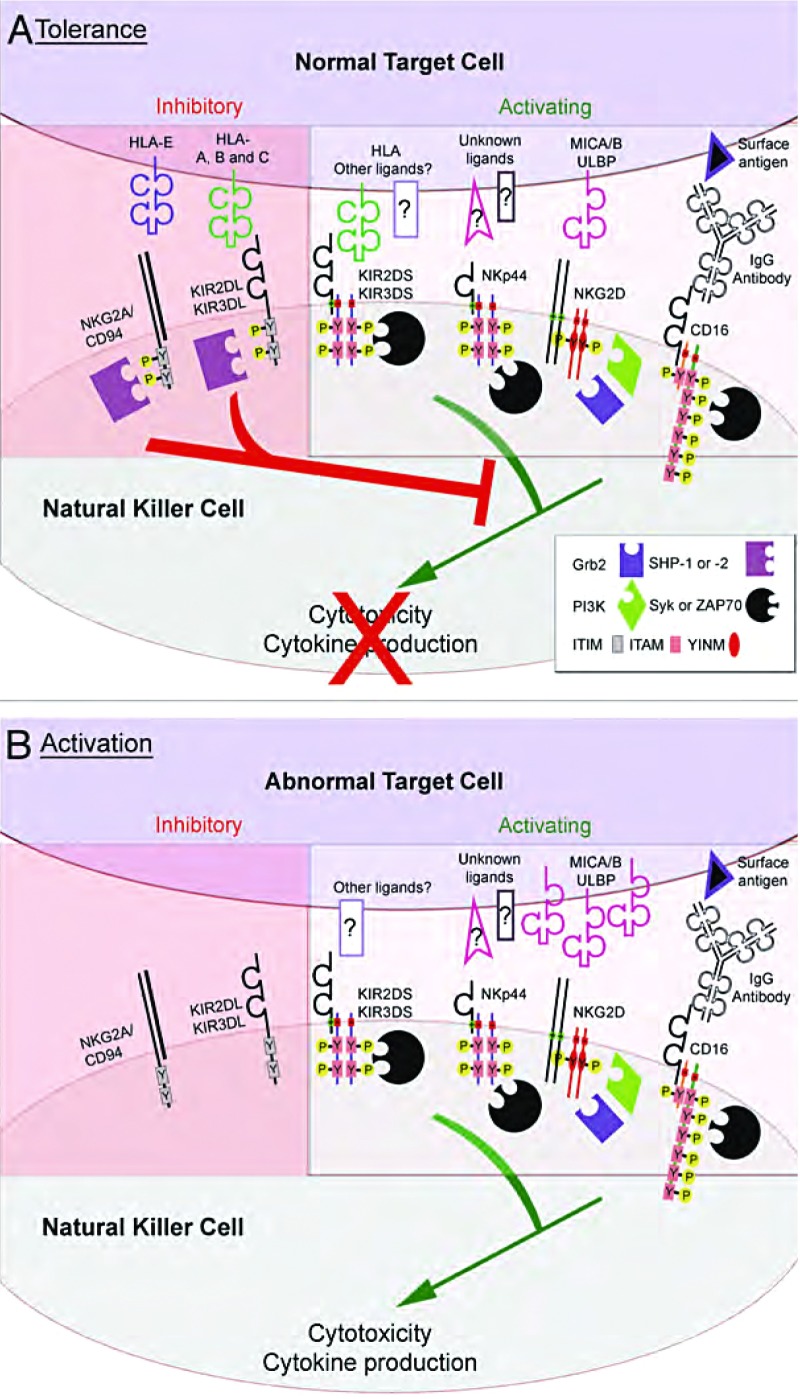
NK细胞的活性由抑制性受体和激活性受体之间的平衡调控。NK细胞的活性是NK细胞与含MHC-Ⅰ的正常靶细胞相互作用的结果（A，耐受）；NK细胞与MHC-Ⅰ表达丢失的异常肿瘤细胞相互作用的结果（B，激活）。激活性受体（KIR、NKG2D、CD16和NCRs，图中仅显示了NKp44）与靶细胞上的配体结合后通过辅助分子的同二聚体或异二聚体的跨膜交换刺激NK细胞发挥细胞毒性和生成细胞因子。这些辅助分子募集信号效应分子[Syk或ZAP-70，磷脂酰肌醇-3激酶（PI3K）或Grb2]至磷酸化酪氨酸（Y-P）胞浆基序（ITAM或YINM），介导下游激活性信号的传导。当细胞发生应激反应、癌性转化或病毒感染时，一些激活性受体的配体（如NKG2D的配体MICA/B和ULBP）表达上调，进一步增强NK细胞活性。通过正常细胞表面的MHC-Ⅰ分子（HLA-A, -B, -C和-E）与抑制性受体结合，正常细胞可逃避NK细胞依赖性细胞毒性作用。与MHC-Ⅰ结合后，抑制性KIR（KIR2DL和KIR3DL）和受体NKG2A/CD94的胞质ITIM序列的酪氨酸残基发生磷酸化。ITIM磷酸化可引起含SH2区域的磷酸酶SHP-1和/或SHP-2的募集，抑制近膜端酪氨酸的磷酸化，进而阻止激活性信号的传导 NK cell activity is controlled by the balance of inhibitory and activating receptors. NK cell activity resulting from the interaction of a NK cell with a normal, MHC-Ⅰ bearing target (A, tolerance) and an abnormal, tumor cell that has lost MHC-Ⅰ expression (B, activation). Engagement of activating receptors (KIR, NKG2D, CD16 and the NCRs; only NKp44 is shown) with ligands on target cells stimulates NK cell cytotoxicity and the production of cytokines through transmembrane charge-based association with homo- or heterodimers of accessory molecules. The accessory molecules recruit signaling effector molecules (Syk or ZAP-70, phosphatidylinositol 3-kinase (PI3K), or Grb2) to tyrosine phosphorylated (Y-P) cytoplasmic motifs (ITAM or YINM), which mediate downstream activation signaling. Some activating receptor ligands are upregulated after cellular stress, cancerous transformation or viral infection (e.g., the NKG2D ligands, MICA/B and ULBP), further increasing NK cell activity. Normal cells are protected from NK cell-dependent cytotoxicity throug the engagement of inhibitory receptors with MHC-Ⅰ molecules (HLA-A, -B, -C and -E) on the normal target cell surface. Upon engagement with MHC-Ⅰ, inhibitory KIR (KIR2DL and KIR3DL) and NKG2A/CD94 receptors become phosphorylated on tyrosine residues within the cytoplasmic ITIM sequences. ITIM phosphorylation leads to the recruitment of SH2 domain-containing phosphatases SHP-1 and/or SHP-2, which dominantly suppressthemembrane-proximaltyrosine phosphorylation events to block activation signaling

相反，可识别MHC-1分子的两种主要类型的NK细胞抑制性受体是“丢失自我”识别能力的分子基础，即：抑制性KIR和异源二聚性NKG2A/CD94受体^[35, 36]^。抑制性KIR可识别经典MHC-1分子的亚型[人白细胞抗原（HLA）-A，-B和-C]，而NKG2A/CD94可识别非经典MHC-Ⅰ分子──HLA-E^[37]^。抑制性KIR和CD94/NKG2A与大多数细胞表面普遍存在的MHC-Ⅰ分子的结合使NK细胞对正常细胞耐受。一旦KIR和NKG2A/CD94与靶细胞表面的MHC-Ⅰ配体结合后，KIR和NKG2A/CD94即可使蛋白酪氨酸磷酸酶募集至细胞质膜，对抗激活性受体信号，从而抑制细胞毒性作用和细胞因子的产生（图 1）^[38-41]^。靶细胞表面配体表达水平的改变可显著影响激活性受体和抑制性受体间的信号平衡，并将改变NK细胞的总活化阈值。以这种方式，MHC-Ⅰ缺陷细胞会缺乏抑制性受体的配体，从而会成为NK细胞介导的攻击靶标。

## KIR的家族成员及其在信号传导中的作用

KIR（即CD158）是一个由1 4个多态性基因（KIR2DL1-5, KIR3DL1-3, KIR2DS1-5, KIR3DS1）编码的受体家族^[35]^，其中7个为抑制性基因，7个为激活性基因（表 1）。尽管现有的综述大多关注于KIR表达对NK细胞功能的影响，但值得注意的是，KIR亦表达于T细胞亚群，包括恒定型（invariant）NKT细胞，因此也可以直接影响它们的功能^[42]^。

**1:** 杀伤细胞免疫球蛋白样受体（KIR）的家族成员 Killer cell Ig-like receptor (KIR) family members

基因名	别名	配基上的识别序列	配基的常见等位基因
KIR2DL1	CD158a, nkat1	HLA-C2	C2: Cw2, Cw4, Cw5, Cw6
KIR2DL2	CD158b1, nkat6	HLA-C1>HLA-C2	C1: Cw1, Cw3, Cw7, Cw8
KIR2DL3	CD158b2, nkat2	HLA-C1>HLA-C2	C1: Cw1, Cw3, Cw7, Cw8
KIR2DL5A^*^	CD158f	Unknown	
KIR2DL5B^*^	KIR2DL5.2	Unknown	
KIR3DL1	NKB1, nkat3	HLA-Bw4 and some HLA-A	B08, B27, B57, A24
KIR3DL2	CD158k, nkat4	Certain HLA-A allotypes	A3, A11
KIR3DL3	CD158z	Unknown	
KIR2DL4	CD158d	HLA-G	
KIR2DS1	CD158h	HLA-C2^A^	C2: Cw2, Cw4, Cw5, Cw6
KIR2DS2	CD158j, nkat5	HLA-C1^A^	C1: Cw1, Cw3, Cw7, Cw8
KIR2DS3	nkat7	HLA-C1^A^	C1: Cw1, Cw3, Cw7, Cw8
KIR2DS4	CD158i, nkat8	disease peptide?; HLA-C^A^	Cw3, Cw4^B^
KIR2DS5	CD158g, nkat9	Unknown	
KIR3DS1	CD158e2, nkat10	HLA-Bw4?	
^*^KIR2DL5 gene is duplicated and encoded by two separate loci within the LRC gene cluster; ^127^ ^A^Activating KIR bind classical HLA molecules with low affinity; ^B^There are conflicting reports as to the HLA-C alleles that KIR2DS4 binds.76, 77 Note: Reprinted with permission from the copyright holder©2009 Landes Bioscience KIR2DL5基因由LRC基因簇内两个单独的位点复制和编码^[127]^; ^A^激活性KIR与经典HLA分子结合的亲和力较低；^B^与KIR2DS4结合的HLA-C等位基因的相关报道不一致。 注：本图得到版权所有者©2009 Landes Bioscience复制许可			

KIR的命名基于其胞外区的结构特征（2D *vs* 3D，代表胞外免疫球蛋白样区域的数目）和胞质尾区的长度（L，长*vs* S，短）。通过胞质区的长度可以推测KIR的功能，L受体通常是抑制性的，S受体均为激活性的^[43, 44]^。该法则的唯一例外是KIR2DL4，它是一个独特的激活性受体，对细胞因子生成的刺激作用很强，但对细胞毒性的刺激作用却较弱^[45, 46]^。抑制性受体包含一个或两个免疫受体酪氨酸抑制基序[ITIM; (I/V)xYxx(L/V)]，是抑制性KIR发挥作用的必要且充分条件^[40, 47]^。当抑制性KIR与靶细胞表面的MHC-Ⅰ结合后，Src家族蛋白酪氨酸激酶使ITIM序列磷酸化，从而暴露出蛋白酪氨酸磷酸酶SHP-1和SHP-2的特异性锚定位点（图 1）^[23, 48]^。SHP-1/2的募集导致经由蛋白酪氨酸激酶转导的激活性受体的信号被显著抑制^[38, 39, 47]^。另一方面，激活性KIR缺乏ITIM，但相应地拥有一段带电荷的跨膜残基，有助于与跨膜辅助蛋白DAP12或FcεRI-γ的物理结合（图 1）^[23, 48, 49]^。DAP12和FcεRI-γ通过位于胞质区的免疫受体酪氨酸激活基序[ITAM; Yxx(L/I/V)x_6-8_Yxx(L/I/V)]传递活化信号，ITAM被Src家族激酶磷酸化并募集Syk/ZAP-70家族蛋白酪氨酸激酶，继而介导下游的活化信号^[23]^。与激活性KIR一样，NCR和CD16与包含ITAM的辅助蛋白（DAP12、FcεRI-γ和TCR-ζ）结合，通过募集Syk/ZAP-70促进活化作用。或许，NKG2D与辅助蛋白DAP10结合，通过募集磷脂酰肌醇激酶-3和Grb2促进活化作用^[50]^。

## KIR的配体

不同的KIR识别不同的人类经典MHC-Ⅰ分子亚类──HLA-A，-B和-C^[4]^。HLA由KIR基因（19q13.4）中位于单独染色体（6p21.3）上的一组基因编码，并因此具有遗传独立性^[51]^。在高等哺乳动物，尤其是灵长类中，KIR很快进化，形成一个高度多态性的受体家族，可识别更多多态性HLA分子众多亚类的共同部分^[52, 53]^。与T细胞受体（T cell receptor, TCR）相似，KIR通过MHC-Ⅰ的肽结合槽与HLA结合；但是，与TCR不同的是，KIR仅与MHC-Ⅰ肽结合槽的羧基末端结合^[54]^。尽管如此，有研究发现不同的肽段可削弱KIR与MHC-Ⅰ的结合，提示某些肿瘤肽段或病毒感染肽段可能会削弱抑制性KIR的识别能力，从而降低NK细胞的活化阈值^[55-57]^。

所有的各种抑制性KIR可识别100%的已知HLA-C的同种异型（allotypes）（可分为C1和C2亚组）及HLA-B和HLA-A的同种异型的亚类。HLA分子中的特定序列部分决定着配体与KIR结合的特异性（表 1和图 2A）。HLA-C2的同种异型的特征是第80位氨基酸为赖氨酸，KIR2DL1可与HLA-C2的同种异型结合^[58-60]^。KIR2DL2/KIR2DL3为同一基因的等位基因，HLA-C1同种异型的第80位氨基酸为天冬酰胺，二者可结合^[21, 61, 62]^。有报道称KIR2DL2与部分HLA-C1同种异型有更高的亲和力，因此有文献显示，在某些情况下，与KIR2DL3相比，KIR2DL2为更强的抑制性受体^[63, 64]^。有报道认为（直接结合试验或功能试验），KIR2DL2/3亦均可与某些HLA-C2同种异型和少数非传统的HLA-B同种异型结合^[63, 65]^。因此，KIR2DL1-3联合起来可抑制NK细胞对表达任一HLA-C同种异型的细胞的细胞毒性作用。KIR3DL1可识别所谓“Bw4”的特定基序，这一基序见于大约40%的已知HLA-B同种异型和某些HLA-A同种异型^[66]^。其余的HLA-B的同种异型以Bw6基序为特征，这一基序不能被KIR3DL1识别^[67-69]^。HLA-Bw4基序可进一步分为第80位氨基酸为异亮氨酸或苏氨酸的同种异型，分别对KIR3DL1呈现高亲和力和低亲和力^[67, 68, 70]^。因此，KIR3DL1仅可抑制NK细胞对表达HLA-B和HLA-A同种异型离散型亚组的靶细胞的细胞毒性作用。已知KIR3DL2仅可识别HLA-A3和HLA-A11同种异型，特定的呈递肽段可调节它们之间的相互作用^[55, 71, 72]^。到目前为止，KIR2DL5和KIR3DL3的配体尚不明了，且有待鉴定。

**2:**
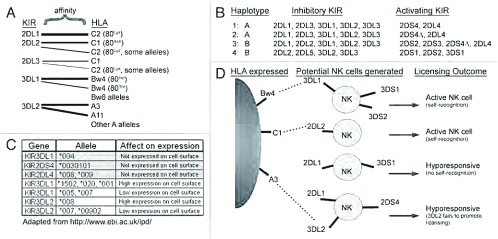
KIR遗传和表达的多因素的多样性。（A）抑制性KIR对不同HLA分子的亲和力不同，但也有小部分重叠。KIR2DL1对HLA-C2亲和力强；KIR2DL2和KIR2DL3对HLA-C1的亲和力强，但对HLA-C2亲和力弱；KIR3DL1可识别HLA-B和某些HLA-A同种异型内的Bw4基序，而非Bw6基序；已知KIR3DL2仅识别HLA-A3和-A11。图中显示了配体间的相互作用，粗线代表亲和力强。没有连接线的HLA分子（如Bw6和其它等位基因A）不能被KIR识别。（B）KIR基因是两种主要的单倍体（单倍体A和单倍体B）基因阵列所遗传的。单倍体A编码大部分的抑制性KIR（KIR2DL/KIR3DL），仅编码1-2个激活性KIR（KIR2DL4和/或KIR2DS4），而更多的单倍体B编码其它的激活性KIR（KIR2DS/KIR3DS）。图B显示了4个假想供者的两种单倍体并列出遗传的抑制性和激活性KIR。由于大部分KIR2DS4基因中22bp的缺失导致提前到达终止密码子（称为KIR2DS4Δ基因），因此很多携带单倍体A和某些单倍体B的个体不能表达功能性KIR2DS4蛋白。（C）小的KIR等位基因多态性可影响KIR在细胞表面的呈递及其在细胞表面的表达水平。很多小的多态性仅仅是一个或数个氨基酸不同，目前还不清楚某些变异是否也会改变KIR对HLA的识别。图中列出了可影响表达的等位基因KIR2DL4、KIR2DS4、KIR3DL1和KIR3DL2。（D）在NK细胞分化过程中，KIR表达的不同形成了KIR表达和NK细胞活性多种多样的NK细胞池。在本例中，如果KIR2DL1为表达于个体NK细胞表面的唯一抑制性受体，那么缺乏HLA-C2表达的人会生成低反应性的NK细胞（不具有自身识别能力）。相反，表达其相应配体存在的抑制性KIR（KIR2DL2, KIR3DL1）的NK细胞是完全具有功能的（具有自我识别能力）。目前情况变得更加错综复杂，Fauriat等的研究显示KIR3DL2并不能给予NK细胞“执照”^[92]^，这提示仅仅表达自我识别受体KIR3DL2的NK细胞也是低反应性的。由于激活性（“S”）KIR与HLA的亲和力较低，它们之间的相互作用未在图中标注 Multifactorial diversity of KIR inheritance and expression. (A) Inhibitory KIR have separate and sometimes overlapping affinity for distinct HLA molecules. KIR2DL1 has strong affinity for HLA-C2, while KIR2DL2 and KIR2DL3 recognize HLA-C1 with strong affinity and HLA-C2 weakly. KIR3DL1 recognizes Bw4 motifs, but not Bw6 motifs in HLA-B and some -A allotypes, while KIR3DL2 is only known to recognize HLA-A3 and -A11. The ligand interactions are indicated, with wider lines signifying a stronger affinity, while the HLA groups lacking lines (e.g., Bw6 and other A alleles) are not recognized by any KIR. (B) KIR genes are inherited in gene arrays named haplotypes of which two major subtypes exist (designated haplotypes A and B). The A haplotypes encode mainly inhibitory KIR (KIR2DL/KIR3DL) with only 1–2 activating KIR (KIR2DL4 and/or KIR2DS4), while the more diverse B haplotypes encode additional activating KIR (KIR2DS/KIR3DS). Representative examples for both haplotypes from four hypothetical human donors are shown with the inherited inhibitory and activating KIR listed. Due to a 22 bp deletion leading to a premature stop codon in a large fraction of KIR2DS4 genes (denoted KIR2DS4Δ), many individuals with A and some B haplotypes fail to express functional KIR2DS4 protein. (C) Minor KIR allelic polymorphism can affect both the presence of KIR on the cell surface and their level of surface expression. Many of these minor polymorphisms vary at only one or a few amino acids and it is currently unclear whether some variations also alter HLA recognition. Examples of some KIR2DL4, KIR2DS4, KIR3DL1 and KIR3DL2 alleles that affect expression are shown. (D) The variegated expression of KIR during NK cell differentiation will generate a pool of NK cells that is heterogeneous for KIR expression and NK cell activity. In this example, a person lacking expression of HLA-C2 would generate a hyporesponsive NK cell if KIR2DL1 was the only inhibitory receptor expressed on an individual NK cell (no self-recognition). In contrast, NK cells that express inhibitory KIR for which ligand is present (KIR2DL2, KIR3DL1), would become fully functional (self-recognition). Adding to the complexity, Fauriat et al. showed that KIR3DL2 was unable to license NK cells, 92 indicating that an NK cell only expressing KIR3DL2 as a self-recognizing receptor would also be hyporesponsive. Due to their low affinity, interactions between activating ("S") KIR and HLA are not indicated

鉴于激活性KIR与抑制性KIR的胞外区间高度的同源性（约99%），许多研究已报道激活性KIR与抑制性KIR可识别同一HLA分子，尽管激活性KIR与HLA分子的亲和力明显较低（表 1）^[58, 64, 73-75]^。然而，由于亲和力较低和某些报道间的不一致，结合的特异性尚不清楚。例如，两项研究表明HLA-C1和-C2均为KIR2DS4的潜在配体^[76, 77]^。与KIR2DS1与Epstein-Barr病毒感染细胞之间的相互作用相似，HLA分子呈递的特定肽段可能增强激活性KIR-HLA的亲和力^[75]^。由于有研究显示KIR2DS4可识别黑色素瘤细胞表面的非MHC-Ⅰ多肽，因此激活性KIR可能还有迥然不同的配体^[78]^。

## KIR的多样性

大多数的HLA多样性源于各遗传基因的序列多态性（遗传的两个拷贝的HLA-A，-B和-C，分别来自父亲和母亲），与HLA不同，KIR多样性是多重原因的（图 2）^[79]^。KIR多样性的多因素特性基于：（1）人类个体遗传的14个现有KIR基因（单体型）的不同的等位基因组合；（2）各KIR基因内存在小的等位基因序列的多态性；以及（3）个体外周血中NK细胞表面的不同KIR分子的表达富于变化^[44]^。

KIR基因位于染色体19q13.4上的白细胞受体复合体中约150 kb的片断内。KIR基因串联排列在单倍体内，它们在单倍体内的数量以及激活性等位基因和抑制性等位基因的比例迥异^[44]^。最常见的人类单倍体为单倍体A，编码大多数的抑制性受体，包括KIR3DL3、KIR2DL2/3、KIR2DL1、KIR3DL1和KIR3DL2，以及激活性受体KIR2DS4和KIR2DL4（图 2B）^[35]^。更重要的是，研究发现在80%的欧洲裔美国人中，KIR2DS4由于小的缺失而丧失功能^[43, 80]^，而且大部分遗传的KIR2DS4等位基因不表达于细胞表面^[81]^。因此，许多单倍体A缺乏激活性KIR的表达。相反，单倍体B的编码基因的总数和激活性KIR基因的数量更加多样化，单倍体B的广义定义为：除KIR2DS4之外，至少还具有一个激活性KIR2DS/KIR3DS基因（图 2B）^[35, 43]^。在不同种族间KIR单倍体A和B的分布各不相同：在欧洲血统的个体中二者的分布大致相等，而在韩国人和日本人中以单倍体A为主，但在澳大利亚土著人中以单倍体B居多^[80, 82-84]^。HLA配体的分布亦可根据种族来归类。例如，与66%的高加索人相比，92%的日本人表达KIR2DL2/3的配体──HLA-C1^[85]^。因此，不同种族表达的KIR与HLA配体的组合差异较大。

除KIR基因单倍体的多样性外，KIR和HLA基因内小的多态性显著影响它们与配体的亲和力与及细胞表面的表达水平（图 2C）。例如，KIR3DL1是多态性最丰富的KIR基因之一，其不同等位基因在细胞表面的表达水平大相径庭^[85, 86]^。某些KIR等位基因编码从不表达于细胞表面的受体（如KIR3DL1^*^004、KIR2DL4^*^008和KIR3DS1^*^049N）；然而，目前尚不清楚这些细胞内受体是否发挥某些作用^[45, 87, 88]^。另外，一些研究发现HLABw4基序外的残基不仅影响与KIR3DL1的亲和力，而且影响KIR3DL1的抑制能力^[70, 89, 90]^。与对HLA-Bw4的这些影响相似，HLA-C1和HLA-C2基序外的残基也分别影响与KIR2DL2/3和KIR2DL1的亲合力^[63]^。因此，等位基因的多态性不仅影响KIR和HLA的亲和力，还影响KIR或HLA在细胞表面的表达水平，而且这一多样性的全面影响尚未完全阐明。

## 外周血NK细胞池中KIR表达的差异

除基因水平的多样性外，KIR在不同NK细胞上有不同的表达模式，因此在外周血中形成表达不同KIR组合的NK细胞库（图 2D）^[79, 91]^。在NK细胞的发育过程中，多种KIR随机表达于NK细胞表面。由于KIR和HLA独立地遗传于不同的染色体上，NK细胞库中某些抑制性KIR与其同源的配体并不协同表达。在NK细胞发育过程中，KIR的表达一旦启动，即会在NK细胞的整个生命周期及其产生的子代细胞中保持稳定^[92-94]^。在NK细胞库中，这种随机表达导致个体的NK细胞或缺乏一个或多个KIR，或表达一个或多个KIR^[92]^。在NK细胞的成熟过程中，NKG2A/CD94二聚体先于KIR表达，并且NKG2A/CD94与KIR的共表达多见于表达较少抑制性KIR的NK细胞^[92]^。

在NK细胞的发育过程中，还有其它因素影响KIR表达的组合方式。Fischer等发现，在NK细胞的分化过程中KIR2DL3先于KIR2DL1表达，并且表达的先后顺序影响KIR表达的总体频率（即，KIR2DL3^+^NK细胞所占的比例大于KIR2DL1^+^NK细胞）^[95]^。由于每个等位基因均被单独调控，故基因拷贝数亦影响总KIR表面表达频率。因此，与仅遗传一个等位基因的人群相比，遗传了特定KIR两个等位基因的人群表达特定KIR的NK细胞池所占比例较大^[85, 96]^。

## KIR/HLA的遗传特性与对包括癌症在内的疾病的易感性

由于KIR与同源性HLA配体之间的相互作用显著影响表达这些受体的NK细胞、T细胞或NKT细胞的总体应答反应，因此这种相互作用可潜在地影响先天性免疫反应和适应性免疫反应^[97, 98]^。而且，由于KIR和HLA配体的编码基因均具有高度多态性，所以不同KIR/HLA基因组合（被称为KIR/HLA复合基因型）的联合遗传可影响对病理状态的敏感性，这或许不足为奇^[98]^。一系列广泛的研究报道不同的KIR/HLA复合基因型与对多种疾病的易感性或耐受性有关，这些疾病包括：病毒感染性疾病（丙肝、HIV）、自身免疫性疾病和慢性炎症性疾病（脉管炎、牛皮癣、糖尿病、特发性支气管扩张和鸟样弹性脉络膜视网膜病变）以及影响妊娠结果的状况（自发性流产和先兆子痫）^[31, 43, 96]^。此外，某些研究报道KIR/HLA复合基因型与对某些癌症（黑色素瘤、白血病、宫颈肿瘤和霍奇金氏淋巴瘤）的易感性有关^[99-103]^。

KIR与HLA基因（或特定的等位基因）的特定组合的联合遗传与对疾病的易感性相关，这提示多种可能的影响机理^[31, 43, 96]^。许多研究发现，抑制性较弱的KIR/HLA复合基因型（如低亲和力KIR2DL3纯合子与同源性HLA-C1配体）与对炎症性疾病的易感性呈正相关。这些研究提示，活化阈值较低（由于抑制作用较弱）导致NK细胞和T细胞的过度反应，这可能会促使免疫系统过度激活。与之相对，已有研究显示抑制性较弱的KIR/HLA复合基因型对某些病毒感染具有保护作用。这些资料提示，NK细胞和溶细胞性T细胞的抑制性KIR间的相互作用越弱，感染清除能力越强。然而，Carrington实验室发现激活性KIR3DS1/Bw4和抑制性KIR3DL1/Bw4的受体/配体组合均对HIV/AIDS具有保护作用（衡量标准为AIDS的进展率和病毒的负荷量）^[104]^，说明KIR/HLA组合对免疫应答反应有多重影响，在进一步了解其机制之前，上述结果难以解释。

具有自我识别能力的KIR/HLA间的相互作用至少以两种方式影响NK细胞的应答反应。第一，相互作用的联合效能可改变激活性信号和抑制性信号间的平衡，抑制性KIR/HLA复合基因型的较强相互作用会导致NK细胞的应答反应降低。这样，KIR作为变阻器来调控NK细胞的活化阈值。就此而论，由于NK细胞对呈递的病毒或肿瘤细胞的应答反应降低，在与感染或癌症对抗时，较强的抑制性KIR/HLA复合基因型可能具有危害性。第二，在被称之为NK细胞教育（education）或执业（licensing）的过程中，具有自我识别能力的KIR/HLA间相互作用的效能亦可影响应答反应成熟NK细胞池的发育在NK细胞发育过程中，具有自我识别能力的MHC-Ⅰ通过抑制性受体来促进NK细胞的最终成熟和功能实现。相反，具有自我识别能力的抑制性受体（KIR或NKG2A）表达缺乏的NK细胞不能正常成熟，且应答反应降低。因此，KIR/HLA间的相互作用，尤其是具有自我识别能力的抑制性KIR/HLA间的相互作用，可潜在影响NK细胞的应答反应:（1）在NK细胞成熟过程中，它们可促进NK细胞功能池的形成；（2）调控成熟NK细胞的活化阈值。KIR/HLA间的相互作用影响成熟NK细胞池应答反应的上述两种机制间存在潜在矛盾。因此，很难预测特定的KIR/HLA复合体基因型是如何影响体内免疫应答反应的。

尽管探寻KIR/HLA对疾病的影响的研究越来越多，但用这些相关研究来诠释KIR/HLA对免疫功能的影响为时尚早。例如，许多已公布的研究尚存在诸多问题：（1）样本量有限（因此缺乏显著的统计意义）；（2）缺乏恰当的对照组（如年龄和种族的匹配，因为年龄与发病有关，而且不同种族间KIR和HLA的分布各不相同）；（3）一些研究在分析时未剔除不表达于细胞表面的KIR等位基因；（4）未在配体的背景下考虑KIR；（5）缺乏有关不同的KIR多态性对HLA配体识别能力的所有影响的全面理解。此外，免疫系统通过不同的方式影响不同疾病，而且KIR/HLA的关系可潜在影响一种疾病的多个阶段（如起始阶段、疾病进展阶段和发病年龄）；因此，很难预知共同的主题来解释KIR/HLA复合体基因型是如何影响不同疾病易感性的。

## KIR/HLA组合对基于造血干细胞的癌症治疗的影响

造血干细胞移植（hematopoietic stem cell transplantion, HSCT）常用于血液系统恶性肿瘤的治疗，帮助清除肿瘤细胞和重建免疫系统^[105]^。与组织移植的其它方式不同，HSCT中的部分HLA同种异型可带来良好的移植物抗肿瘤作用，以治疗血液系统恶性肿瘤，尽管移植物抗宿主病（graft-versus-host disease, GVHD）为副作用。近年来，有研究显示，供者的抑制性KIR与受者的HLA配体间的同种异型可进一步影响HSCT治疗血液系统恶性肿瘤。

鉴于个体间KIR和HLA基因的丰富的多样性及在发育过程中个体NK细胞上KIR表达的多样化，在HSCT后产生的某些供者源性NK细胞可能对缺乏恰当HLA配体（称之为KIR/HLA配体错配）的受者组织产生“同种异型反应”^[105-107]^。因此，在HSCT的过程中，移植供者表达的KIR与移植受者表达的HLA配体之间的错配可能会产生同种异型反应性NK细胞池，该细胞池作用于受者组织及肿瘤细胞。有研究显示，KIR-HLA配体错配尤其可获益于“单倍体相合的”HSCT，在“单倍体相合的”HSCT中供者干细胞与受者干细胞的HLA单倍体仅部分匹配。父母或兄弟姐妹一般是合适的且常易于获得的单倍体相合的供者。

2002年，Ruggeri等发现，同种异体反应性NK细胞通过促进移植物抗肿瘤反应延长生存期并减少复发，显著改善单倍体相合的HSCT对急性髓系白血病（AML）患者的治疗^[108]^。有趣的是，他们还发现，NK细胞的同种异体反应性降低了HSCT最严重的副作用——GVHD的发生率，GVHD由同种异型的供者源性T细胞介导。与之前的报道一致，研究者进一步证实，GVHD的减少是由于移植受者体内残余的抗原呈递细胞的清除^[108, 109]^。值得注意的是，可能是由于正常细胞极少表达激活性受体的配体，同种异型NK细胞似乎并不直接介导HSCT受者中GVHD对正常组织的反应。

不幸的是，其它研究小组的初步实验结果未能重现KIR-HLA错配对HSCT疗效的有利影响^[110-112]^。但是，这些最初的追踪研究采用与Ruggeri等明显不同的HSCT治疗方案。随后的一些研究^[107, 113, 114]^发现，为实现KIR/HLA配体错配在单倍体相合的HSCT治疗中的有利效果，某些治疗方案的联合至关重要：（1）对受者行侵入性清髓和免疫抑制的预处理，如全身放疗和化疗，以减少移植排斥和降低癌症负荷；（2）移植前消除HSC中的大量T细胞，以防止发生GVHD；以及（3）输注大量的HSC。

对单倍体相合的HSCT疗效显著有利的另一因素是，供者外周血中有可能存在大量对受者产生同种异型反应的NK细胞（部分NK细胞缺乏可识别受者HLA的抑制性KIR）^[65, 114]^。有研究显示，HSCT受者体内的逐步成熟的NK细胞池呈现与供者相似的KIR表达谱^[115, 116]^。因此，将供者体内的大部分同种异型NK细胞移植给受者可能更为有益。Fauriat等（2008年）最近发现，潜在供者间的NK细胞池中同种异型反应性NK细胞所占比例各不相同（02%-62%），这提示并非所有的个体均为合适的HSCT的供者^[92, 106]^。许多研究小组的研究均实施过直接输入成熟的同种异型NK细胞，且显示该方法是安全的，并具有一定的抗白血病作用^[117]^。此项技术的未来发展具有治疗前景。

除了对单倍体相合的HSCT有潜在益处外，KIRHLA的关系亦影响其它HSCT的效果。例如，Hsu等发现AML和骨髓增生异常综合征患者在接受抑制性KIR/HLA配体错配的HLA相合的HSCT后无疾病生存期延长^[118]^。相似的是，Leung等的研究显示，各种淋巴瘤和实体瘤患者在接受自体HSCT（在介入化疗后重新输入患者的HSC）治疗后抑制性KIR/HLA配体错配有益于病人生存期的延长^[119]^。与之相对，Cooley等的最新研究发现，AML患者在接受富T细胞的HSCT后未能获益于抑制性KIR/HLA配体错配^[120]^。但是，此研究显示，当供者含有至少KIR的一个单倍体B时（单倍体B比单倍体A含有更多的激活性KIR基因），受者的无复发生存期显著改善，这提示在这些背景下移植的NK细胞表达更多的激活性KIR也可能是有利的^[120]^。

总之，许多报道显示，在HSCT疗法治疗AML和其它癌症中KIR介导的NK细胞的同种异型反应性的作用是有利的。但是，KIR/HLA配体错配而导致的同种异型反应非常复杂，而且至今机制未明。未来的工作需侧重于改善移植条件，并提高分子水平对KIR/HLA组合收益的理解。

## 癌症治疗的KIR治疗策略

由于抑制性KIR在调节NK细胞活化中发挥重要作用，因此用以减弱KIR功能的治疗策略可能强化NK细胞在治疗癌症和病毒感染中的活性^[121]^。这与仍表达HLA分子的肿瘤患者的治疗尤其密切相关，因这些肿瘤并非NK细胞攻击的靶标。除了与抑制性KIR的功能相关外，SHP-1和SHP-2磷酸酶是各种组织中一系列的抑制性受体的共同信号转导效应分子，因此如果应用药物抑制这些磷酸酶，可能不会有特异治疗性仅仅增强体内NK细胞的功能。或许，治疗性特异性阻滞抑制性KIR对配体的识别可能会降低NK细胞的活化阈值。

最近，Binyamin等采用体外自体原代NK细胞/转化靶细胞模式系统发现，抗体介导的KIR阻滞显著促进CD16依赖性ADCC反应^[122]^。重要的是，这些体外研究显示，单一的抑制性KIR阻滞不能显著提高对自体转化细胞的杀伤力，这提示其它的自我耐受机制可阻止对无抗体靶向的肿瘤细胞的攻击。相似的是，最近应用KIR/HLA转基因小鼠模型进行的体内研究发现，抗体介导的KIR阻滞剂可提高对肿瘤细胞的杀伤力并降低自我反应性^[123]^。而且，Binyamin等应用体外自体靶向细胞系统发现，对纯合子低亲和力CD16等位基因（FcγRIIIA 158F）的供者NK细胞，KIR抑制剂可更好地强化ADCC反应^[122]^。这一点非常重要，因为滤泡淋巴瘤患者的低亲和性FcγRIIIA等位基因（158F *vs* 158V）为纯合子，所以患者对利妥昔单抗治疗的临床反应率较低^[34, 124, 125]^。另一项新近的研究描述了一种新开发的人抗体，在人源化小鼠的研究中发现，该抗体可阻断KIR2DL1-3，且可显著提高AML急变期的NK细胞毒性和ADCC反应^[126]^。综上，在癌症患者中，KIR的阻滞有可能成为切实可行的治疗选择以促进NK细胞介导的对肿瘤的细胞毒性反应，这些报道为其提供了临床前依据。

## 结论

总之，我们对KIR/HLA间相互作用多样性的理解在不断深入，但新的发现也不断地显示其复杂性。然而，越来越明确的是：这些高度多态性的受体和其众多的配体可改变免疫功能，从而影响许多疾病（包括癌症）的易感性，以及HSCT治疗白血病（尤其是AML）的有效性。未来研究应阐明细胞表面表达KIR和HLA的不同多态性变异体以及受体/配基的亲和力对免疫功能的影响，并明确KIR的其它配体。此外，研究特定的KIR/HLA的相互作用如何影响NK细胞的成熟和功能，从而影响某些个体的健康。其机制有待更多的研究作深入探讨。另外值得注意的是，KIR的某些效应可能是通过作用于T细胞亚群介导的。最后，如何选择特定的KIR/HLA组合以优化造血干细胞移植治疗癌症的疗效，有待其它研究来明确。

## Acknowledgments

We thank Drs. Jennifer Rhodes (FCCC) and Wesley Rose (Arcadia University, Glenside, PA) for constructive criticism during manuscript preparation. This work was supported by R01 grants CA-083859 and CA-100226 (K.S.C.), training grant CA009035-32 (A.K.P.) and partially by Centers of Research Excellence grant CA06927 (FCCC) from the National Institutes of Health. The research was also supported in part by an appropriation from the Commonwealth of Pennsylvania. Its contents are solely the responsibility of the authors and do not necessarily represent the official views of the National Cancer Institute.
